# Factors Influencing the Risk of Major Amputation in Patients with Diabetic Foot Ulcers Treated by Autologous Cell Therapy

**DOI:** 10.1155/2022/3954740

**Published:** 2022-04-11

**Authors:** J. Husakova, R. Bem, V. Fejfarova, A. Jirkovska, V. Woskova, R. Jarosikova, V. Lovasova, E. B. Jude, M. Dubsky

**Affiliations:** ^1^Diabetes Centre, Institute for Clinical and Experimental Medicine, Prague, Czech Republic; ^2^First Faculty of Medicine, Charles University, Prague, Czech Republic; ^3^Second Faculty of Medicine, Charles Unviersity, Prague, Czech Republic; ^4^Transplant Surgery Department, Institute for Clinical and Experimental Medicine, Prague, Czech Republic; ^5^Diabetes Center, Tameside and Glossop Integrated Care NHS Foundation Trust and University of Manchester, Lancashire, UK

## Abstract

**Introduction:**

Autologous cell therapy (ACT) is one of the last options for limb salvage in patients with chronic limb-threatening ischemia (CLTI) and diabetic foot ulcers (DFU). However, some patients may still undergo a major amputation even after ACT, but the risk factors for this are not known. Therefore, the aim of our study was to assess the risk factors for major amputation in patients with CLTI and DFU during a 2-year follow-up after ACT.

**Methods:**

One hundred and thirteen patients after ACT were included in our study and divided into two groups: Group 1 with major amputation (AMP; *n* = 37) and Group 2 without amputation (nAMP, *n* = 76). The risk factors for major amputation were evaluated before ACT and included factors relating to the patient, the DFU, and the cell product.

**Results:**

The AMP group had significantly higher C-reactive protein (CRP) levels compared to the nAMP group (22.7 vs. 10.7 mg/L, *p* = 0.024). In stepwise logistic regression, independent predictors for major amputation were mutation of the gene for methylenetetrahydrofolate reductase (MTHFR) with heterozygote and homozygote polymorphism 1298 (OR 4.33 [95% CI 1.05-17.6]), smoking (OR 3.83 [95% CI 1.18-12.5]), and CRP > 10 mg/L (OR 2.76 [95% CI 0.93-8.21]). Lower transcutaneous oxygen pressure (TcPO_2_) values were observed in AMP patients compared to the nAMP group at one month (24.5 vs. 33.2, *p* = 0.012) and at 3 months (31.1 vs. 40.9, *p* = 0.009) after ACT.

**Conclusion:**

Our study showed that the risk for major amputation after ACT in patients with CLTI and DFU is increased by the presence of MTHFR heterozygote and homozygote gene mutations, smoking, and higher CRP at baseline. Lower TcPO_2_ at one and 3 months after ACT may also have a predictive value. Therefore, it is necessary to stop smoking before ACT, treat any infection, and, above all, consider antiaggregation or anticoagulant treatment after the procedure.

## 1. Introduction

Diabetic foot ulcers (DFU) represent a late complication of diabetes and result in delayed healing and often to minor or major amputation and are associated with higher morbidity and mortality [1–3]. A meta-analysis of 16 studies reported that the 5-year mortality rate after major amputation in patients with diabetes and PAD was 62.2% [4].

The main factor leading to amputation is delayed wound healing in patients with diabetes. Poor healing of DFU is influenced by hyperglycaemia, chronic inflammation, micro- and macrovascular dysfunction, and neuropathy [5, 6]. Other factors for impaired healing are immunological abnormalities such as disruption of macrophage function, altered function of keratinocytes and fibroblasts, and decreased stem cell homing [2, 7, 8].

One of the most important factors that influence ulcer healing is tissue perfusion. Chronic limb-threatening ischemia (CLTI) represents the end stage of peripheral arterial disease with high mortality and morbidity, increased rates of major amputation, and decreased quality of life [9]. PAD affects almost 20% of older Americans (above 60 years). The risk factors were hyperlipidemia, hypertension, diabetes, chronic kidney disease, and smoking. Firnhaber and Powell described the risk of PAD to be ten times higher in patients with at least three of these factors [10]. Levels of circulating omentin-1 have been proved to be associated with the severity of PAD [11].

Cigarette smoking is a major risk factor for cardiovascular disease, stroke, and mainly PAD [12]. Tobacco use also has a strong correlation with CLTI [13, 14].

One of the factors influencing tissue perfusion is hemocoagulation disorders [15, 16]. Acquired abnormalities of the coagulation cascade and inherited thrombophilia in patients with diabetes can be a predictive factor for thrombosis [17]. Methylenetetrahydrofolate reductase (MTHFR) with polymorphism A1298C (MTHFR A1298C) may be associated with high levels of homocysteine and increases the risk of cardiovascular disease [18].

Another factor that contributes to impaired wound healing and reduced tissue oxygenation is infection. Acute inflammatory marker C-reactive protein (CRP) helps to diagnose infection at an early stage [19]. The presence of raised CRP and reduced lower limb perfusion significantly decreased wound healing in diabetic patients with CLTI [20]. CRP and increased proinflammatory cytokines interleukin 6 correlated with worse outcomes after endovascular procedure in patients with diabetes and PAD [21].

To reduce amputations in patients with CLTI and DFU, additional methods are needed to improve tissue oxygenation. Autologous cell therapy (ACT) represents an important therapeutic role in CLTI in whom revascularization is not an option (no-option CLTI, NO-CLTI) [22]. The goal of ACT is to promote the growth of collateral vessels through neovascularization and arteriogenesis. Nowadays, it is reserved only for NO-CLTI because in accordance with international guidelines, we should always prefer standard revascularization procedures if possible and to reserve cell therapy only for patients in clinical trials [9]. The cost of this procedure is usually comparable to that of PTA with stenting. ACT may reduce amputation rate, rest pain, and tissue loss and lead to a decrease in mortality and an increase in amputation-free survival [23]. At our centre, we have extensive experience in the use of ACT in management of CLTI and DFU [24]. Although we have shown that this treatment reduces the risk of amputation, we cannot prevent this complication in some patients. Therefore, we decided to analyse the factors that may increase the risk of amputation even after ACT.

The aim of our study was to assess the factors that increase the risk of major amputation in patients with CLTI and DFU during a 2-year follow-up after ACT.

## 2. Methods

One hundred and thirteen patients with diabetes type 1 or 2, CLTI, and chronic foot ulcer that were not suitable for standard revascularization and treated in our foot centre during the last 13 years were included in the study and treated with ACT. For the purposes of this study, patients were divided into two groups (Group 1 (*n* = 37) with major amputation (AMP) and Group 2 (*n* = 76) without amputation (nAMP)) and followed up for 2 years after ACT. Demographic and baseline characteristics of both groups are shown in Table 1.

Exclusion criteria for ACT were as follows: deep infection in the foot, limb edema, untreated advanced diabetic retinopathy requiring laser therapy or retinopathy with high risk of retinal bleeding, severe hematological disease and deep vein thrombosis, myocardial infarction or stroke in the last 6 months, neoplastic process of any organ, and life expectancy less than 6 months.

The CLTI was evaluated by angiography, ultrasound, computed tomography, or magnetic resonance angiography of the lower limb and by transcutaneous oxygen pressure (TcPO_2_) of less than 30 mm Hg at baseline (measured by Radiometer TCM400, Radiometer Medical ApS, Brønshøj, Denmark). Patients with CLTI and DFU were consented for ACT after discussion with interventional radiologists, vascular surgeons, and foot care specialists.

All factors that might potentially increase risk for amputation were assessed at baseline and before ACT and were divided into three groups: first group was patient-related factors such as patient's age, body mass index, smoking history, diabetes duration and diabetes control, and laboratory results such as CRP, number of leucocytes, renal function, serum lipids, coagulation parameters (levels of protein S, protein C, fibrinogen, and homocysteine), and thrombophilic mutations (factor V Leiden, prothrombin mutation MTHFR C677T and A1298C). The second group included ulcer characteristics (size and depth, presence of infection, edema, and limb perfusion, presence of ischemia and gangrene, and value of TcPO_2_). The third group of factors was related to the cell product that included cells' viability, number of leukocytes, and CD34+ cells (Table 1).

Foot ulcers were classified by WIfI score assessing the size of the wound, presence of infection and/or ischemia, and by other classification systems focused on foot ulcers, ischemia, and infection (PEDIS, TEXAS, and Wagner; Table 2). The presence of local inflammation was confirmed if any of the following were present: purulence, erythema, tenderness, warmth, or induration with limitation to the skin and tissue [25].

We have described the ACT method in detail in our previous publication [24]. Briefly, the ACT was obtained from bone marrow from the iliac crest using a Jamshidi needle under local or general anesthesia. The separation of mononuclear fraction was performed either by Harvest Smart PReP2 (Harvest Technologies Corporation, Plymouth, MA, USA) or by succinyl gelatin (Gelofusine; B. Braun, Melsungen, Germany) which accelerated erythrocyte sedimentation.

The final suspension, a volume of 40-60 mL, was injected intramuscularly in the ischemic lower limb in a series of 40–50 punctures injecting 1 mL at each site and keeping 1-2 cm distance between them on both sides of the gastrocnemius muscle, deep into the soleus muscle and into the dorsal and plantar muscles and also into the edges of the wound.

Patients were followed up at intervals of 1, 3, and 6 months and after 1 and 2 years after therapy. At baseline and at each follow-up visit, TcPO_2_ was measured. The effect of ACT was evaluated by ulcer healing and changes in TcPO_2_ and compared to baseline values. All patients were checked up biweekly or monthly and received best local wound care with regard to their type of wound (iodium-based solutions for gangrene or wet healing dressings for chronic nonhealing ulcers); in some cases for deep wounds, a negative pressure wound therapy was used.

## 3. Statistical Analysis

Statistical analysis was done using BMDP Statistical Software Inc. 8.1 (Medcalc, Ostend, Belgium) and Medcalc version 17.8.6. We used the *χ*^2^ test, stepwise logistic regression, and calculated univariate odds ratios (ORs), with 95% confidence intervals (CIs). The figures were performed by GraphPad Prism 7.0.4 (GraphPad Software, La Jolla, CA, USA).

## 4. Results

**Figure 1:**
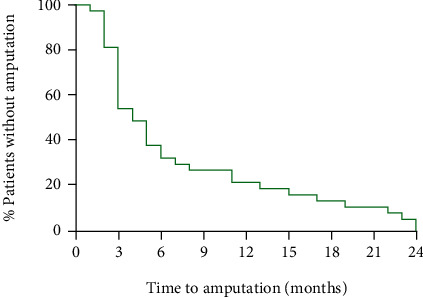
Time to amputation in the AMP group (*n* = 37).

Of the 113 patients included in the study, 37 underwent major amputation and 76 did not have an amputation during the 2-year follow-up. There was no difference in patients' characteristics between the amputated and nonamputated groups at baseline (Table 1). The time to major amputation in the AMP group is shown in Figure 1. The classifications of DFU at baseline and up to 6 months in both groups are shown in Table 2.

**Figure 2:**
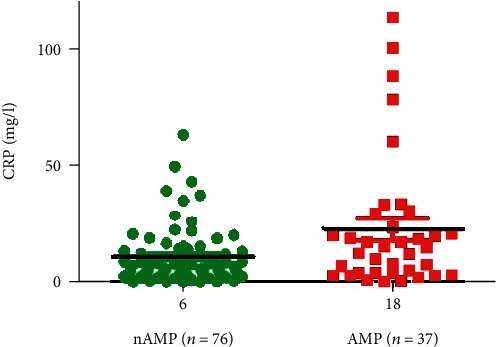
Baseline CRP in the nAMP and AMP groups.

In a stepwise logistic regression, the independent predictors for amputation after ACT were MTHFR A1298C mutation, previous or present smoking history, CRP > 10 mg/L at baseline, and a decrease in TcPO_2_ 1 month after ACT (Table 3). The most important predictor for major amputation (OR 4.33 [95% CI 1.05-17.6]) was MTHFR mutation with homo- and heterozygous polymorphism A1298C. Our results also confirmed tobacco use as a significant risk factor of major amputation almost 4 times in smokers compared to ex-smokers (OR 3.83 [95% CI 1.18-12.5]). CRP represented a risk factor with levels higher than 10 mg/L (OR 2.76 [95% CI 0.93-8.21]). CRP levels in the AMP group were higher in comparison with those of the nAMP group (22.7 vs. 10.7 mg/L, *p* = 0.024; Figure 2).

**Figure 3:**
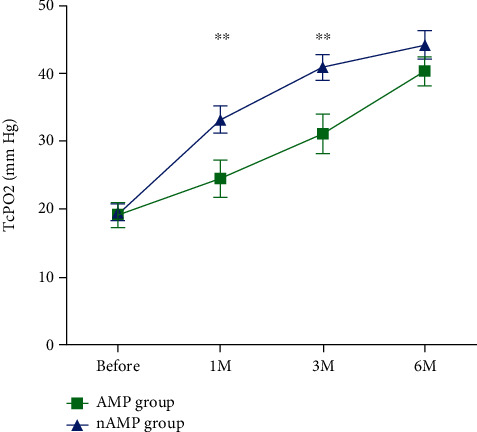
TcPO_2_ in the AMP and nAMP groups up to 6 months after ACT. ∗∗ represents a significant difference between the AMP and nAMP groups in 1 month and 3 months.

Lower TcPO_2_ values were observed in AMP patients compared to the nAMP group at one month (24.5 vs. 33.2, *p* = 0.012) and at 3 months (31.1 vs. 40.9, *p* = 0.009) after ACT (Figure 3). The results also showed that a decrease in TcPO_2_ by 1 mm Hg increased the risk of major amputation by 4% (OR 0.959; 95% CI 0.926-0.993).

We observed the presence of multiresistant bacteria in the wound swabs (methicillin-resistant Staphylococcus aureus, Proteus sp., Klebsiella sp.) in both groups without a significant difference between them (Table 1). The WIfI scores (AMP 3.8 vs. nAMP 3.7, NS) and SINBAD scores (AMP 4.1 vs. nAMP 4.1) were not different between the AMP and nAMP groups at baseline (Table 1).

## 5. Discussion

This is the first study to report on amputation after ACT. We have shown that the main major amputation risk factors in patients with DFU and CLTI treated by ACT were MTHFR A1928C mutation, smoking, elevated CRP, and decrease in TcPO_2_. Many of these factors have been associated with amputations in other studies, but have not been evaluated after ACT [26–28].

In our study, the incidence of major amputations over the 2 years after ACT was 32.7%; however, majority of the patients underwent an amputation in the first 6 months (Figure 1). The incidence of major amputation in patients with diabetes and CLTI in the literature is very high (23-72%) [9, 29]. Kalbaugh et al. observed in a retrospective population-based analysis incidence of major amputation in 72% of patients with CLTI and predominantly in diabetic patients in 61% within 16 years [29].

Risk factors for amputation have been studied previously. The risk of major amputation was, for example, higher in men, people with neuropathy, foot ulcers with higher Wagner score, patients with worse diabetes control, with higher leucocytes, thrombocytes, C-reactive protein, and decrease in HDL-cholesterol, albumin, C-peptide, uric acid, and ankle-brachial index below 0.8 [26–28]. Prior minor amputation increased the risk of subsequent major amputation ten times and almost twentyfold increased risk of minor reamputation [27].

In our study, in contrast to previous studies, we demonstrated the predictive value of TcPO_2_ measured at 1 month after ACT for major amputation. TcPO_2_ is a commonly used indicator of the effect of ACT on improving tissue perfusion [30]. We believe that our study could contribute to more usage of TcPO_2_ to assess the effect of ACT in upcoming studies, because TcPO_2_ directly evaluates microcirculation (and therefore can assess the formation of new collaterals) and usually is more definitive and less variable than the laser Doppler flowmetry. The increased value of TcPO_2_ may indicate the revascularization effect of cell therapy and improvement in limb perfusion. We assume that both values are important—rate of TcPO_2_ increase and the absolute value of TcPO_2_—at the end of the follow-up period, but we do not suppose that there is an absolute TcPO_2_ threshold that could predict the major amputation after ACT because amputation could be influenced by other factors such as osteomyelitis, cellulitis, or unbearable pain.

Another factor that is not usually listed among the risks for major amputation is inherited thrombophilia. An association of thrombophilic mutations with other pathological conditions has been published. For example, an association of the polymorphism MTHFR A1298C was strongly associated with the presence of end-stage renal disease [18]. MTHFR A1298C is found in 7 to 12% of North American, European, and Australian populations [31]. In our study, we observed substantially higher prevalence of this mutation (38% in nAMP group and 61% in AMP group). Gemmati et al. demonstrated the interaction between MTHFR homozygotes and the prevalence of and also the increased risk for both arterial and venous thromboses [32]. Lupi-Herrera et al. observed a higher risk of arterial and venous thromboembolic disease and described an increase in massive and submassive pulmonary embolism and acute myocardial ischemia in patients with MTHFR A1298C (*p* = 0.017) and increased homocysteine levels [33]. In our study, we did not show a clear association of major amputations with higher homocysteine levels, although there is a pathogenetic link between the MTHFR mutation and homocysteine [31]. Homocysteine is an intermediate amino acid containing a sulfhydryl group that comes from the methylation of methionine. The accumulation of homocysteine leads to pathologies such as rheumatoid arthritis, cancer, and vascular occlusive disease, and it is used also as a coronary artery disease risk factor [33, 34]. Hyperhomocysteinemia in association with accelerated atherosclerosis is an independent risk factor for cardiovascular, cerebrovascular, and peripheral artery disease. Homocysteine induces endothelial dysfunction and vascular inflammation [35].

Another important predictor of major amputation in our study was tobacco use. Smoking increased the risk of major amputation in patients after ACT almost four times (OR 3.83). The risk of PAD increases after 30 years of smoking, with cardiovascular disease after 20 years, and intense smoking of more than 20 cigarettes daily increased the probability of subclinical PAD in comparison with lower intensity use [36, 37]. In patients who stop smoking, the risk would return to the level of nonsmokers after 10 years of smoking cessation [36]. Kianoush et al. observed an 8-fold increased risk of PAD, with ankle-brachial index less than 1.0, and also an increase in aortoiliac calcium by almost tenfold in smokers [37].

Another independent predictor for major amputation after ACT was CRP above 10 mg/L. Chronic inflammation with elevated CRP has been shown to be associated with PAD [9]. In studies evaluating DFU and osteomyelitis in people with diabetes, it was observed that CRP above 35 mg/L had a sensitivity of 80% and specificity of 89% for the presence of diabetic foot infection [38]. A higher CRP level in patients with CLTI has been associated not only with increased risk of major amputation but was also with higher mortality and poorer prognosis [39].

Our study showed no significant differences between groups in LDL cholesterol, but in both groups the values of LDL-c were above the recommended levels. All included patients met the criteria of high risk of proatherosclerotic changes and cardiovascular disease, but at baseline, it did not meet the recommended levels of 1.8 mmol/L of LDL cholesterol (AMP 2.25 ± 0.74 vs. nAMP 2.4 ± 0.98) [40].

The DFU classification was without significant differences between the AMP and nAMP groups because patients included in our study had advanced diabetic complications such as neuropathy and CLTI and most of them were classified in the higher grades of WIfI and other classification systems. Their chronic ulcers were either deep and colonised with bacteria or gangrenous that worsened the prognosis of the wound (Table 1).

Therefore, we feel that the findings from our study could help with selection of the patients for ACT. To maximize the success of ACT, we should instruct patients to stop smoking and increase their adherence with DFU treatment and to aggressively treat infection before the ACT procedure. Test of MTHFR gene prior to ACT could possibly prevent later thrombotic complications after the procedure by early indication of antithrombotic treatment. On the other hand, this test is very expensive and the prothrombotic status is also dependent on the levels of homocysteine [31].

## 6. Study Limitations

All patients who included in the study had severe PAD with no revascularization possibilities and as mentioned were NO-CLTI patients. ACT was performed as the last treatment option before considering limb amputation. The results of the study may be affected by the lower number of patients in the groups. The indication for amputation was made on the basis of a group decision of experts and the patient's opinion; however, a more subjective bias cannot be excluded. The nature of the study meant that a control group could not be included. Some of the baseline data could not be influenced such as smoking history, long-term glycaemic control, or LDL cholesterol levels.

## 7. Conclusion

The results of our study showed that the risk of major amputation in patients with diabetes after ACT with no-option CLTI is increased by inherited thrombophilia—MTHFR A1298C gene mutations, smoking, and higher CRP levels before ACT treatment. The decrease in TcPO_2_ 1 month after ACT may also have a predictive value for major amputation. Therefore, for these patients, it is necessary to stop smoking and before ACT treat any diabetic foot infection. After ACT, it is advisable to monitor the level of TcPO_2_ and, above all, consider adequate antiaggregation or anticoagulant treatment after the procedure mainly in patients with thrombophilic mutations.

## Figures and Tables

**Table 1:** Baseline characteristics of patients.

	Amputation (AMP) (*n* = 37)	Without amputation (nAMP) (*n* = 76)	*p*
Sex			
Male	30 (81%)	64 (84%)	NS
Female	7 (19%)	12 (16%)	
Age (years)	66 ± 13.7	67 ± 10.5	
Cholesterol (mmol/L)	4.0 ± 0.9	4.2 ± 1.1	
LDL cholesterol (mmol/L)	2.3 ± 0.74	2.4 ± 0.98	
Body mass index (kg/m^2^)	27.8	26.7	
Malnutrition	8 (22%)	15 (20%)	
Diabetes mellitus			
Diabetes type 1	5 (14%)	19 (25%)	NS
Diabetes type 2	32 (86%)	57 (75%)	
HbA1c (mmol/mol)	57 ± 16.7	58.7 ± 13.5	
Duration of diabetes (years)	25.6 ± 13.2	24.9 ± 12.1	
CRP (mg/L)	22.7 ± 28	10.7 ± 12	*p* = 0.024
Comorbidities			
Chronic kidney disease (CKD)	2.43	2.63	NS
Modification of diet in renal disease study equation (MDRD)	0.74	0.95	
Chronic heart failure	26 (70%)	41 (54%)	
Smoking			
Smoker	5 (13%)	9 (12%)	NS
Ex-smoker	21 (57%)	22 (29%)	
Years of history of smoking	23 ± 20	18 ± 18	
Nonsmoker	11 (30%)	45 (59%)	
Treatment			
Acetylsalicylic acid	22 (59%)	34 (45%)	NS
Clexane	2 (5%)	6 (8%)	
Clopidogrel	5 (14%)	12 (16%)	
Rivaroxaban	0 (0%)	5 (7%)	
Dabigatran	2 (5%)	12 (16%)	
Warfarin	6 (16%)	7 (9%)	
Statins	25 (68%)	37 (49%)	
Vascular interventions			
Percutaneous transluminal angioplasty (PTA) (number of patients (%)/number of procedures)	35 (95%)/1.62	29 (38%)/1.74	NS
Bypass (number of patients (%)/number of procedures)	13 (35%)/0.59	25 (33%)/0.36	
Cultivation			
MRSA or ESBL infection	5 (14%)	7 (9%)	NS
Another ATB-resistant infection	6 (16%)	23 (30%)	
Osteomyelitis	6 (16%)	23 (30%)	
Osteomyelitis			
Osteomyelitis in X-ray	11 (29.7%)	33 (43.4%)	NS
Positive probe to bone test	6 (16%)	17 (22%)	
Prothrombophilic factors			
MTHFR A1298C	22 (61%)	29 (38%)	NS
Homocysteine (*μ*mol/L)	12.07	16.51	
MTHFR C677T	13 (35%)	31 (41%)	
Homocystein (*μ*mol/L)	12.80	15.40	
Protein C (%)	105.9 ± 29.1	104 ± 29.6	
Protein S (%)	90.7 ± 31.8	98.8 ± 34.3	
Cell product			
Viability (%)	96.2	93.9	NS
Total CD34+ in product (∗10^6^)	12.6	13.9	
Total leucocytes in product (∗10^9^)	2.6	2.2	

**Table 2:** Classification systems of DFU at baseline and at all follow-up visits.

	AMP				nAMP			
	Baseline (*n* = 37)	1 M (*n* = 35)	3 M (*n* = 29)	6 M (*n* = 14)	Baseline (*n* = 76)	1 M (*n* = 76)	3 M (*n* = 72)	6 M (*n* = 71)
Wagner (mean)	3.43	3.40	3.14	2.93	2.87	2.79	1.78	1.06
Wagner 3 (%)	6 (16%)	8 (23%)	8 (28%)	3 (21%)	7 (9%)	9 (38%)	5 (7%)	4 (6%)
Wagner 4 (%)	24 (65%)	21 (60%)	11 (38%)	4 (29%)	33 (43%)	30 (12%)	12 (17%)	4 (6%)
PEDIS infection	1.54	1.91	2.07	2.00	0.68	0.58	0.22	0.08
WIfI (mean)								
Wound	1.81	1.91	2.00	1.93	1.93	1.84	1.74	0.86
Ischemia	2.95	2.49	2.31	1.93	2.97	2.20	1.74	1.48
Foot infection	0.57	0.91	1.07	1.00	0.64	0.47	0.19	0.07
TEXAS (%)								
TEXAS B only infection	0 (0%)	1 (3%)	5 (17%)	1 (7%)	0 (0%)	0 (0%)	2 (3%)	2 (3%)
TEXAS C only ischemia	28 (76%)	14 (40%)	3 (10%)	4 (29%)	49 (64%)	33 (43%)	26 (36%)	20 (28%)
TEXAS D infection and ischemia	9 (24%)	17 (49%)	16 (55%)	5 (36%)	27 (36%)	27 (36%)	4 (6%)	1 (1%)

**Table 3:** Independent predictors for major amputation.

Factor	OR	95% CI
MTHFR A1298C	4.33	1.05-17.6
Smoking	3.83	1.18-12.5
CRP > 10 mg/L	2.76	0.93-8.21
TcPO_2_ at 1 month	0.959	0.926-0.993

## Data Availability

The data will be uploaded together with the manuscript. The data used to support the findings of this study are included within the supplementary information file in the folder Supplementary_Information_files_KB_all_amputation_JoDR_SI.
